# Khat use and psychotic symptoms in a rural Khat growing population in Kenya: a household survey

**DOI:** 10.1186/s12888-019-2118-3

**Published:** 2019-05-07

**Authors:** Linnet Ongeri, Fredrick Kirui, Erastus Muniu, Veronica Manduku, Leah Kirumbi, Lukoye Atwoli, Safari Agure, Peter Wanzala, Lydia Kaduka, Mercy Karimi, Richard Mutisya, Elizabeth Echoka, Joseph Mutai, David Mathu, Charles Mbakaya

**Affiliations:** 1 Kenya Medical Research Institute, Centre for Clinical Research, P.O. Box 54840 00200, Off Mbagathi Road, Nairobi, Kenya; 2 Kenya Medical Research Institute, Centre for Public Health Research, P.O. Box 54840 00200, Off Mbagathi Road, Nairobi, Kenya; 3 Moi University School of Medicine, P.O Box 4606, Eldoret, 30100 Kenya; 4 Rongo University College, School of Physical Sciences, Technology & Engineering, P.O. Box 103-40404, Rongo, Kenya

**Keywords:** Khat, Psychotic symptoms, Sub Saharan Africa, Kenya, And household survey

## Abstract

**Background:**

Khat is an amphetamine like psychostimulant chewed by over 10 million people globally. Khat use is thought to increase the risk of psychosis among its chewers. The evidence around this however remains inconclusive stemming from the scanty number of studies in this area and small study sample sizes. We undertook a large household survey to determine the association between psychotic symptoms and khat chewing in a rural khat growing and chewing population in Kenya.

**Methods:**

For this cross-sectional household survey, we randomly selected 831 participants aged 10 years and above residing in the Eastern region of Kenya. We used the psychosis screening questionnaire (PSQ) to collect information on psychotic symptoms and a researcher designed sociodemographic and clinical questionnaire to collect information on its risk factors. We used descriptive analysis to describe the burden of khat chewing and other substance use as well as rates and types of psychotic symptoms. Using a univariate and multivariate analyses with 95% confidence interval, we estimated the association between khat chewing and specific psychotic symptoms.

**Results:**

The prevalence of current khat chewing in the region was at 36.8% (*n* = 306) with a male gender predominance (54.8%). At least one psychotic symptom was reported by 16.8% (*n* = 168) of the study population. Interestingly, psychotic symptoms in general were significantly prevalent in women (19.5%) compared to men (13.6%) (*p* = 0.023). Khat chewing was significantly associated with reported strange experiences (*p* = 0.024) and hallucinations (*p* = 0.0017), the two predominantly reported psychotic symptoms. In multivariate analysis controlling for age, gender, alcohol use and cigarette smoking, there was a positive association of strange experiences (OR, 2.45; 95%CI, 1.13–5.34) and hallucination (OR, 2.08; 95% C.I, 1.06–4.08) with khat chewing. Of note was the high concurrent polysubstance use among khat chewers specifically alcohol use (78.4%) and cigarette smoking (64.5%).

**Conclusions:**

Psychotic symptoms were significantly elevated in khat users in this population. Future prospective studies examining dose effect and age of first use may establish causality.

## Background

*Catha edulis*, commonly known as khat, is a psychostimulant plant used by over 10 million people daily, mainly in eastern Africa and the Middle East [1]. Historically, khat was recognized as a ceremonial plant in Kenya, chewed primarily by older men during special social gatherings [2]. Recently however, the pattern of khat consumption has been slowly changing with not only a rise in use among women and the youth but also an increase in quantity of khat chewed [3, 4]. In addition to its longstanding traditional value, the sale of khat is an important contributor to Kenya’s economy. Khat is currently Kenya’s highest valued horticultural export accounting for about 60% of the total value of such exports [5]. It is mainly cultivated in the Eastern part of Kenya and its use remains legal in the country.

Khat is primarily ingested by chewing. The leaves and stalks of the khat plant are slowly chewed to release its juice which is then ingested with saliva. The residual pulp is subsequently pushed to the side of the cheek and the mouth continuously filled with fresh leaves. The accumulated ball of residue is eventually spat out at the end of the chewing session [6]. In addition to its euphoric acute effect, khat users also commonly report increased levels of alertness, decreased hunger and fatigue and a general sense of wellbeing during chewing [6–8]. The euphoric effect is felt within 1 h of chewing khat. Cathinone which is the main alkaloid constituent of khat reaches peak plasma levels 1.5–3.5 h after the onset of chewing [9, 10]. Its average elimination half-life is four and half hours [11]. The acute effects of euphoria and alertness are later proceeded by excessive feelings of anxiety and irritability, poor concentration and insomnia following chewing [12]. The tolerance level of khat is low and withdrawal symptoms linked to khat use include nightmares, tremors, depression, sedation and hypotension [13, 14]. Chronic khat use has additionally been linked to cardiovascular, digestive, genitourinary, endocrine and oral dental health problems [15–19].

On average 100 g of fresh khat leaves contain 36mgs of cathinone, 120 mg cathine and 8 mg norephedrine [20]. Khat is chewed for its psychoactive effect is thought to come from two phenylalkylamines; cathinone and cathine, which are structurally related to amphetamine [21]. Despite notable progress having been made in identifying the pharmacological basis of Khat there is divided opinions on the potency and subsequent neurobehavioral effects. Khat’s effect on the central nervous system is thought to act like amphetamine by increasing the activity of the dopaminergic and noradrenergic transmission. [22]. Previous studies have shown associations between heavy khat use with psychosis and mania [23]. In addition, some studies have pointed towards khat exacerbating or triggering primary psychotic disorders such as schizophrenia [24, 25]. Khat induced psychosis has been described as transient in nature and characterized majorly by symptoms of paranoia and mania [26–28]. However, this research is non-conclusive and primarily based on case reports [29]. Another limitation to previous khat research is the number of neurobehavioral studies focused on highly traumatized migrant populations making findings inconclusive as to whether the trauma or the khat triggered the psychosis [30]. A deeper understanding of the epidemiology of khat associated psychosis is warranted to contribute to the evidence base necessary in developing policies around this plant. In addition, previous local community based studies have shown relatively high rates of participants endorsing at least one psychotic symptom (13.9%) [31], being able to tease out the burden of psychotic symptoms attributable to khat use as compared to other risk factors is key to understanding khat use pathophysiology. Khat remains a controversial plant with differing opinions on its benefits and negative impact on human health [32], hence as part of the Kenyan government’s efforts to understand the burden of khat use and its effects on human health we were tasked to conduct a large, population based study in a rural non migrant population of mainly khat growers. We report here findings on the prevalence of khat use and its association with psychotic symptoms in the survey population.

## Methods

### Study design

We used a cross sectional study design to collect data from randomly selected households.

### Study population and setting

We conducted this study in Meru and Embu County situated in the Eastern Part of Kenya. The two counties border each other and together occupy close to 10,000 km^2^. These are the largest khat growing regions in Kenya and only second to Ethiopia in Africa.

The population of Embu County is estimated at 543,221 while Meru County population currently stands at 1,400,000. The two communities are agrarian with a focus on cash crop cultivation this primarily being khat. The khat variant grown in Embu is referred to as *Muguka* while the Meru variant is known as *Miraa*. The two variants are physically different with the shoots for *Muguka* and the leaves for *miraa* being chewed.

In Embu County, we identified two sub-counties (Mbeere North & Mbeere South) as having high production of *Muguka*, while in Meru, we selected four sub-counties (Igembe North, Igembe Central, Igembe South and Tigania East) for their high production of *Miraa*.

Data collection was carried out between the months of September 2015 to December 2015.

### Participant recruitment

#### Sampling of clusters

Sub-areas (villages) which are the smallest administrative units were the Primary Sampling Unit (PSU) or cluster in this survey. Using the local administration, we listed all sub-areas growing khat in the identified sub-counties.

We then allocated the number of clusters required for each county (Meru and Embu) using income generated annually as a proxy measure and applying square root allocation method. For sub-counties, we determined the number of clusters required using the square root allocation method based on number of sub-areas in the sub-county growing khat.

In each of the Sub-counties, we used a two-stage cluster sampling methodology where the first stage involved selection of required clusters for adults and children using systematic random sampling. In the second stage we used systematic random sampling to uniformly select 10 households from a listing of households in each cluster.

From identified adult clusters, we selected the household head and the lead spouse. From identified child clusters, among the children aged 10–17 years in each selected household, we randomly selected one child by balloting if there were more than one eligible child. In the occasion a household member was found to be unavailable during the first visit, at least two follow up visits on two separate occasions were made to maximize on response. Whenever there was complete failure to get the selected household member, it was captured as a non-response.

### Sample size

Using the Fischer et al. formula and a desired precision of 5% for the indicator with 95% confidence, we required a minimum sample size of 385. Applying a design effect of 2 due to cluster sampling and a non-response rate of 20% we determined a sample size rounded off to 1000.

### Ethics and consent

We were granted scientific approvals; SSC number 2888 from the KEMRI Scientific Review Committee (SSC) and the Scientific and Ethical Review Unit (SERU). We then obtained permission to access the study sites from the relevant authorities.

At the household level we sought informed consent from all the adult participants prior to the interview. For participants aged less than 18 years, informed consent was obtained from either their parents or guardians followed by assent by the participant. We informed participants that participation in the study was voluntary and information collected during the study was solely for study purpose. Participants signed written informed consent after detailed explanation of the study purpose. All consents and study documents were in both English and Kiswahili translations. We asked recruited participants to pick the language they preferred for the interview. Trained research assistants conducted interviews in secluded areas to maintain privacy of participants. For participants who required medical attention, we referred them for appropriate medical care.

### Variables

All study questionnaires were forward translated into the local language (Kiswahili) and then back translated into English. Pretesting and piloting of the study tools was undertaken in a different locality from selected clusters for face and content validity. This ensured the content was understandable to the study population (adults and children). Piloting procedure involved administration of the tools by the study clinicians/nurses followed by interviews with participants about their opinions, concerns and challenges about questions asked. Data was collected using a structured face to face interview format using either English or Kiswahili translations depending on the participants’ preference.

We used a researcher designed questionnaire to collect data on socio-demographic characteristics of participants as well as specific khat use behaviour. A separate simplified questionnaire was used for children to collect sociodemographic and khat use behaviour.

We used Alcohol, Smoking and Substance Involvement Screening Test (ASSIST) to collect information on other substance use [33].

We then assessed the presence of five types of psychotic symptoms (mania/hypomania, thought control, paranoia, strange experience, and hallucinations) occurring in the past year using the Psychosis Screening Questionnaire (PSQ) [34] . In some instances, to determine the severity of the symptoms we used one follow-up questions after the main probe question for each type of psychotic symptom.

The questions and response options required for the endorsement of each psychotic symptom were as follows:

- Mania/hypomania: over the past year, have there been times when you felt very happy indeed without a break for days on end? (yes)
- Was there an obvious reason for this? (no)
- Thought control: over the past year, have you ever felt that your thoughts were directly interfered with or controlled by some outside force or person? (yes)
- Did it come about in a way that many people would find hard to believe, for instance, through telepathy? (yes)
- Paranoia: over the past year, have there been times when you felt that people were againstyou? (yes)
- Have there been times when you felt that people were deliberately acting to harm you or your interests? (yes)
- Strange experience: over the past year, have there been times when you felt that something strange was going on? (yes)
- Was it so strange that other people would find it very hard to believe? (yes)
- Hallucination: over the past year, have there been times when you heard or saw things that other people could not? (yes)

We diagnosed probable psychosis when at least one of the five experiences was selected.

### Statistical analysis

We carried out data analysis using IBM SPSS version 21.0 statistical software and R software. Analysis included descriptive statistics (means, standard deviations & medians for continuous variables, proportions and frequency distributions for categorical variables). We confirmed distribution characteristics using Kolmogorov-Sminorf test and Exploratory Data Analysis (EDA) for all the continuous data. Student T-test (for normally distributed data) and Mann-Whitney U test (for skewed data) tested for differences in continuous variables between categories of dependent variables. For categorical variables Chi-square and where applicable, we used Fisher’s exact probability tests. A *p*-value of < 0.05 was considered statistically significant. To determine association of psychotic symptoms and khat chewing while controlling for effect of other variables we carried out binary logistic regression analysis.

## Results

After sampling 650 households from 65 clusters (26 in Embu and 39 in Meru) we enrolled a total of 843 participants out of an expected 1000 (313 from Embu and 530 from Meru) resulting in a non-response rate of 16%. Of these 831 completed the PSQ questionnaire with adults making 66.3% (*n* = 551) of the participants.

### Distribution of socio-demographic characteristics & substance use by sex

The median age of participants was 30 years [Interquartile range (IQR) 15–42 years] and ranged from 10 to 78 years.

Over half of the study participants were farmers below 35 years of age and with a lower level of education. Gender comparisons found more males reporting substance use. See Table 1.

**Table 1:** Distribution of socio-demographic characteristics & substance use by sex

Parameter	Total	Sex, %		*P*-value
		Male	Female	
Age group in years	(*N* = 829)	(*N* = 395)	(*N* = 434)	
10–17	33.8	31.6	35.7	< 0.001
18–35	28.3	22.0	34.1	
36–50	23.6	26.3	21.2	
> 50	14.2	20.0	9.0	
Education (Adults only)	(*N* = 549)	(*N* = 271)	(*N* = 278)	
Up to primary	84.7	80.8	88.5	0.017
Secondary	12.2	16.2	8.3	
Tertiary	3.1	3.0	3.2	
Occupation (Adults only)	(*N* = 540)	(*N* = 265)	(*N* = 275)	
Formal & Self employed	13.7	17.7	9.8	0.024
Farmer	79.4	75.1	83.6	
Casual, domestic & other	6.9	7.2	6.5	
Income (Adults only)	(*N* = 460)	(*N* = 256)	(*N* = 204)	
< 5000	35.0	23.0	50.0	< 0.001
5000- < 50000	59.8	69.1	48.0	
50000 & over	5.2	7.8	2.0	
Khat use	(*N* = 831)	(*N* = 396)	(*N* = 435)	
Chewers	36.8	54.8	20.5	< 0.001
Non chewers	63.2	45.2	79.5	
Tobacco use (Daily or almost daily)	(N = 829)	(N = 395)	(N = 434)	
Yes	13.4	26.3	1.6	< 0.001
No	86.6	73,7	98.4	
Alcohol use (Daily or almost daily)	(N = 829)	(N = 395)	(N = 434)	
Yes	3.7	6.3	1.4	< 0.001
No	96.3	93.7	98.6	

### Distribution of socio-demographic characteristics, alcohol and tobacco use by khat chewing

The prevalence of life time use of khat was 44.6% (371 out of 831) while that of current use was 36.8% (306 out of 831). There was a significant gender difference in khat use with 54.8% of males and 20.4% of the females reporting khat use (*p* < 0.001). The rates of khat chewing in children aged between 10 and 17 years was 13.6%. Khat chewing was significantly associated with male sex (*p* < 0.001), younger age (less than 35 yrs) (*p* < 0001), higher level of income (*p* < 0.001) and comorbid alcohol (*p* = 0.001) and tobacco use (p < 0.001). No significant association was seen with level of education and occupation. See Table 2.

**Table 2:** Distribution of socio-demographic characteristics, alcohol and tobacco use by Khat chewing

Parameter	Khat chewing		*P*-value
	n	%Chew	
Sex			
Male	396	54.8	< 0.001
Female	435	20.5	
Age group in years			
10–17	280	13.6	< 0.001
18–35	235	50.6	
36–50	196	46.9	
> 50	118	48.3	
Education (Adults only)			
Up to primary	465	49.2	0.107
Secondary	67	50.7	
Tertiary	17	23.5	
Occupation (Adults only)			
Formal & Self employed	74	60.8	0.068
Farmer	429	46.4	
Casual, domestic & other	37	51.4	
Income (Adults only)			
< 5000	161	41.0	< 0.001
5000- < 50000	275	55.6	
50000 & over	24	79.2	
Tobacco use (Daily or almost daily)			
Yes	111	78.4	< 0.001
No	718	30.5	
Alcohol use (Daily or almost daily)			
Yes	31	64.5	0.001
No	798	35.8	

### Pattern of khat chewing according to gender

Forty-four percent of the participants had attained the age of 15 years when they first started chewing khat, however of note is that more females (32.6%) reported starting to chew khat under 10 years of age compared to males (12.6%). Nearly half of the khat users reported daily use of khat and chewing between one to three bunches of khat in a sitting. Many (76.7%) had chewed khat over a 10 year duration. There was a statistically significant difference in the distribution pattern of khat use between males and females based on age at first use (*p* < 0.001), frequency (*p* = 0.016), amount (p = 0.001) and duration of khat chewing (p < 0.001). See Table 3.

**Table 3:** Pattern of khat chewing by gender

Characteristic	Gender, %		Total (N = 306)	*P*-value
	Male (*N* = 217)	Female (*N* = 89)		
Age when started khat chewing				
Under 10 years	12.6	32.6	18.3	< 0.001
Between 10 and 15 years	39.5	32.6	37.5	
above 15 years	47.9	34.9	44.2	
Khat chewing pattern				
One day per week	3.3	9.8	5.2	0.016
2–3 days per week	26.3	37.8	29.6	
4–6 days per week	16.3	13.4	15.5	
Everyday	54.1	39.0	49.8	
Bunches chewed in a sitting				
Less than one	23.0	42.0	28.5	0.001
Between 1 and 3	49.8	47.7	49.2	
Between 3 and 5	10.6	6.8	9.5	
More than 5	16.6	3.4	12.8	
Number of years chewing khat				
< than 1 year	–	–	–	< 0.001
1–4 years	9.3	29.1	15.0	
5–10 years	7.9	9.3	8.3	
> 10 years	82.8	61.6	76.7	

### Distribution of socio-demographic characteristics by psychotic symptoms

The overall rate of at least one psychotic symptom in the study population was 16.7%. Females had a significantly higher rate of reporting compared to males (*p* = 0.023). The age group of young adults reported significantly higher rates (p < 0.001) with paranoid symptoms featuring predominantly (15.7%). See Table 4.

**Table 4:** Distribution of socio-demographic characteristics by psychotic symptoms

Parameter	N	Mania/ Hypomania	Thought control	Paranoia	Strange experiences	Hallucinations	Any symptom
		%	%	%	%	%	%
Sex							
Male	396	2.5	3.8	7.3	3.0	4.3	13.6
Female	435	3.2	4.4	11.5	5.5	7.1	19.5
P-value		0.551	0.673	0.041	0.079	0.080	0.023
Age group							
10–17	280	2.5	1.8	3.2	1.4	2.5	10.4
18–35	235	5.1	7.2	15.7	6.8	9.8	26.0
36–50	196	1.5	4.1	8.2	4.6	4.6	13.8
> 50	118	1.7	3.4	14.4	5.9	7.6	18.6
P-value		0.105	0.020	< 0.001	0.019	0.003	< 0.001
Education (Adults only)							
Up to primary	465	3.4	5.6	13.8	6.0	7.5	21.1
Secondary	67	1.5	4.5	9.0	6.0	7.5	16.4
Tertiary	17	0	0	0	0	5.9	5.9
P-value		nv	nv	0.151	nv	0.968	0.225
Occupation (adults only)							
Formal & Self employed	74	2.7	2.7	6.8	4.1	8.1	14.9
Farmer	429	3.3	5.4	13.8	6.3	7.2	20.5
Casual, domestic & other	37	2.7	10.8	13.5	5.4	8.1	34.3
P-value		nv	nv	0.248	nv	0.951	0.423
Income (Monthly income for adults only)							
< 5000	161	5.0	6.2	17.4	5.6	6.2	22.4
5000- < 50000	275	2.9	5.5	10.5	6.9	8.0	20.4
50000 & over	24	0	4.2	8.3	4.2	0	8.3
*P*-value		0.334	0.899	0.095	0.781	0.302	0.282

*nv* Chi-square not valid, * = Fisher’s Exact Probability

### Distribution of substance use by psychotic symptoms

Both hallucinations and strange experiences were significantly associated with khat chewing *P* value 0.0017 and 0.024 respectively. Khat users had higher rates of all symptoms (although not significant) except mania/hypomania, which is a mood component. Excluding mania/hypomania symptoms, khat users still had higher rates of overall psychosis at 16.7% compared to 12.8% for non-users with no significant difference (*p* = 0.12). Daily use of tobacco was significantly associated with thought control, paranoid and hallucination symptoms of psychosis while daily use of alcohol was significantly associated with almost all symptoms of psychosis except manic/hypomanic symptoms. See Table 5. A total of 23 (2.8%) participants were taking other psychotomimetic substances (cannabis 10; inhalants 7; sleeping pills 1; heroin/morphine/pain medicine 4; and a combination of cannabis, sleeping pills & hallucinogens 1). Statistical evaluation of use of any of the psychotomimetic substances and psychotic-like symptoms of participants, did not show significant associations.

**Table 5:** Distribution of substance use by psychotic symptoms

Parameter	N	Mania/ Hypomania	Thought control	Paranoia	Strange experiences	Hallucinations	Any symptom
		%	%	%	%	%	%
Khat use							
Yes	306	2.6	5.6	10.5	6.5	8.2	18.6
No	525	3.0	3.2	9.0	3.0	4.4	15.6
P-value		0.719	0.104	0.476	0.017	0.024	0.262
Tobacco use (Daily or almost daily)							
Yes	111	5.4	8.1	16.2	9.9	9.0	27.0
No	718	2.5	3.5	8.5	3.5	5.3	15.2
P-value		0.089^a^	0.028^a^	0.010	0.005^a^	0.119	0.002
Alcohol use (Daily or almost daily)							
Yes	31	3.2	12.9	22.6	12.9	19.4	32.3
No	798	2.9	3.8	9.0	4.0	5.3	16.2
P-value		0.605^a^	0.034^a^	0.022^a^	0.041^a^	0.007^a^	0.019

*nv* Chi-square not valid, ^a^ = Fisher’s Exact Probability

Sex, age group, current Khat chewing, tobacco and alcohol use were subjected to binary logistic regression using backward stepwise (LR) method for strange experiences as well as hallucinations and following tables shows the variables that were retained in the models

### Logistic regression analysis for strange experiences and hallucinations in relation to Khat use

Adjusting for age, sex and tobacco use, the odds of reporting strange experiences was elevated in Khat users at OR, 2.45; 95%CI, 1.13–5.34. Similarly, khat use was associated with higher odds of experiencing hallucinations (OR, 2.08; 95% C.I, 1.06–4.08) controlling for sex, age and alcohol use. See Table 6.

**Table 6:** Results of Logistic regression analysis for Strange experiences and Hallucinations

Variable	Categories	β	S.E.(β)	*p*-value	AOR^a^	95% CI for AOR	
						Lower	Upper
Strange experiences							
Sex	Female	−0.752	0.231	< 0.001	5.828	2.211	15.362
	Male	Reference					
Khat use	Yes	−0.568	0.324	0.024	2.453	1.126	5.340
	No	Reference					
Tobacco use	Yes	1.048	0.292	0.001	5.903	2.095	16.636
	No	Reference					
Constant		−4.935	0.520	< 0.001	0.007		
Hallucinations							
Sex	Female	1.025	0.368	0.005	2.787	1.356	5.728
	Male	Reference					
Age group in years	10–17	Reference					
	18–35	1.044	0.462	0.024	2.840	1.148	7.023
	36–50	0.367	0.531	0.489	1.444	0.510	4.089
	Over 50	1.082	0.535	0.043	2.950	1.033	8.420
Khat use	Yes	0.733	0.343	0.033	2.080	1.062	4.077
	No	Reference					
Alcohol use	Yes	1.492	0.518	0.004	4.446	1.612	12.261
	No	Reference					
Constant		−4.470	0.499	< 0.001	0.011		

^a^*AOR* Adjusted Odds Ratio

## Discussion

The lifetime prevalence of khat use in the study participants was 44.6%. This study found a high overall prevalence of current khat use (36.8%). This prevalence is comparable to a study in Ethiopia reporting a 37.8% prevalence of current khat use [35] but lower than rates in recent population based study in Yemen (80%) [24, 36]. Khat is thought to be a cash crop in this study area, our study findings however show that khat is not only grown for sale but also locally consumed by the residents. Similar to previous khat studies our study found significant gender differences with khat use predominantly being reported in males [37, 38]. Of note is that in our study setting khat is culturally chewed primarily by men. Few studies have reported on khat use in children, though the rates of chewing in children in our study were only 13.6%; low compared to studies earlier done in Yemen [36], it still is a worrisome figure as earlier initiation of substance use is linked to increased risk of drug related morbidity [39, 40].

The overall rates of at least one psychotic symptom reported were slightly higher (16.7%) compared to a local household based study conducted in Western Kenya (13.9%) [31]. Varying levels of risk factors particularly exposure to substance use may explain the difference seen. Interestingly however, unlike similar population based khat studies elsewhere [41], we found a significantly higher rate of psychotic symptoms reported in women compared to men; a finding that was similarly observed in a Western Kenya population survey study [31] . Because khat is predominantly chewed by men it is assumed that men would present with higher levels of psychiatric morbidity including psychosis compared to women which is not the case here. One theory could perhaps be a difference in vulnerability to the exposure to khat however, this is unlikely in our study as reported psychotic symptoms were significantly higher in women compared to men regardless of their use of khat. Previous studies have demonstrated an increased stress sensitivity in women compared to men [42, 43]. Of note is that greater stress sensitivity has been significantly associated with increases in positive psychotic symptoms among females [44]. This could offer one explanation to the higher rates of psychotic symptoms seen in women compared to men in our study. This higher reporting of psychotic symptoms among females than males is in congruent to findings of the Western Kenya study that examined psychotic symptoms in a general population. Though we did not measure life events in the present study, exposure to life events measured in the Western Kenya study found a significant association between experiencing a life events and reporting of psychotic symptoms. When life events were analysed by sex in the study, 4 individual life events namely serious illness, injury or assault to self, bullying, violence at home and running away from your home were associated with increased risk of having psychotic symptoms in females, while none of the same or other life events were associated with increased risk of having psychotic symptoms in males. This finding further supports the theory of gender difference in stress reactivity.

Regarding psychotic symptom profile, khat using participants were more likely to report experiencing strange events and hallucinations compared to other psychotic symptoms. Other studies have linked khat associated psychosis with mania and paranoid symptoms two symptoms that were not statistically significant in this study [45]. It is worth mentioning that a number of studies have continually shown a high rate of concurrent substance use among khat users [46, 47]. Evidenced also in this study with high rates of concurrent cigarette and alcohol use. Polysubstance use may impact on khat related psychosis symptom profile [48, 49]. Alcohol related psychosis for example is primarily characterized by hallucinations while stimulants more commonly induce paranoid symptoms of psychosis [50, 51].

After combining all psychotic symptoms no significant association was seen with psychosis for khat users. However, when looking at individual psychotic symptoms both the unadjusted and adjusted analysis, khat use was associated with elevated odds of reporting strange experiences and hallucinations. Based on the cross-sectional nature of our study design it is difficult to conclude on what came first, whether khat itself caused psychosis or whether the participants with existing psychotic disorders used khat in an attempt to alleviate psychotic symptoms. Further, as there was no time line provided for the psychotic experiences and khat use in the interview questions, it is possible that khat users experienced these psychotic symptoms only when they were under influence of khat (acute intoxication) and not in a more persistent way. In this study, we set out to look at discrete psychotic symptoms rather than to make diagnoses of psychotic disorders. The psychosis screening items on the PSQ were used for this purpose since the instrument has been used in our setting in the past [31], and these are the same questions that would be used to screen for psychotic symptoms in a clinical setting, whether children or adults. Additionally, the items are similar to those in a validated adolescent screening [52]. The strength of this study is the relatively large population size recruited using random sampling techniques. However, our study was conducted in a primarily rural population in eastern Kenya and hence generalizability to other populations is unknown.

## Conclusion

This is the first Kenyan population-based study focusing specifically on khat use. Controlling for alcohol and tobacco use we found psychotic symptoms of hallucinations and strange experiences to be significantly elevated in khat users compared to non-users in this study population. There is need for more epidemiological studies with different study designs to see if similar findings are replicated.

## Abbreviations

ASSIST: Alcohol, Smoking and Substance Involvement Screening Test
EDA: Exploratory Data Analysis
IQR: Interquartile range
KEMRI SSC: Kenya Medical Research Institute Scientific Review Committee
PSQ: Psychosis Screening Questionnaire
PSU: Primary Sampling Unit
SERU: Scientific and Ethical Review Unit
SPSS: Statistical Package for Social Sciences
